# The rules of the game: interprofessional collaboration on the intensive care unit team

**DOI:** 10.1186/cc2958

**Published:** 2004-10-08

**Authors:** Lorelei Lingard, Sherry Espin, Cathy Evans, Laura Hawryluck

**Affiliations:** 1 Associate Professor, Department of Pediatrics and The Wilson Centre for Research in Education, University of Toronto, Ontario, Canada; 2 Nurse Research Fellow, The Wilson Centre for Research in Education, University of Toronto, Ontario, Canada; 3 Assistant Professor, Department of Physical Therapy, University of Toronto, Ontario, Canada; 4 Assistant Professor, Department of Medicine, University of Toronto, Ontario, Canada

**Keywords:** collaboration, conflict, interdisciplinary communication, medical care team

## Abstract

**Background:**

The intensive care unit (ICU) is a nexus for interspecialty and interdisciplinary tensions because of its pivotal role in the care of the hospital's most critically ill patients and in the management of critical care resources. In an environment charged with temporal, financial and professional tensions, learning how to get results collaboratively is a critical aspect of professional competence. This study explored how team members in the ICU interact to achieve daily clinical goals, delineate professional boundaries and negotiate complex systems issues.

**Methods:**

Seven 1-hour focus groups were conducted with ICU team members in two hospitals. Participants consisted of four nursing groups (*n* = 27), two resident groups (*n* = 6) and one intensivist group (*n* = 4). Interviews were audio-recorded, anonymized and transcribed. With the use of a standard qualitative approach, transcripts were analyzed iteratively for recurrent themes by four researchers.

**Results:**

Team members articulated their perceptions of the mechanisms by which team collaboration was achieved or undermined in a complex and high-pressure context. Two mechanisms were recurrently described: the perception of 'ownership' and the process of 'trade'. Analysis of these mechanisms reveals how power is commodified, possessed and exchanged as team members negotiate their daily needs and goals with one another.

**Conclusion:**

Our data provide a non-idealized depiction of how health care professionals function on a team so as to meet both individual and collective goals. We contend that the concept of 'team' must move beyond the rhetoric of 'cooperation' and towards a more authentic depiction of the skills and strategies required to function in the competitive setting of the interprofessional health care team.

## Introduction

Interprofessional tensions can threaten the delivery of quality health care in a hospital setting. Such tensions have been documented in several clinical domains including internal medicine [1-3], pediatric wards [4,5], the operating room [6-8] and the intensive care unit (ICU) [9]. The ICU in particular is a nexus for interspecialty tensions because of its pivotal role in the care of the hospital's most critically ill patients and in the management of critical care resources. Within the hospital community, the ICU exists at the high-stakes intersection of emergency, surgery, internal medicine and palliative care, an intersection where the patient care resources are expensive, in scarce supply and a source of intense competition.

Repeated calls have been made for improved collaboration, communication, congruence and equity within health care teams as ways of improving quality of care and protecting patient safety. Current notions of team-building advocate increasing flexibility in team structure, abolishing hierarchies and cultivating shared decision making [10-16]. Although these are important concepts, they can reflect a naive sense of the team as a unified entity rather than as a collection of individuals with distinct professional identities based on different models of care, skills, economic circumstances and political agendas.

To foster optimal team function, we first need to understand better the forces governing the interactions between professions (for example, nurses and physicians) and between specialties (for example, the ICU team and external consultants) as they work together in an environment charged with professional, temporal and financial tensions. Previous work by our research group has described team dynamics in the ICU [9]. We found that the level of collaboration or conflict within the ICU team, and between the ICU and other specialties, fluctuated on the basis of six key catalysts: authority, education, patient needs, knowledge, resources and time. These findings provided insight into the divisive forces present even in high-functioning teams, and alerted us to the strategies that team members enact as they seek to balance individual needs with team goals.

We also found that 'team', in the ICU, is not a unified body but rather is a complex and fluid entity composed of core and expanded groups. Membership in these groups is continually negotiated on the basis of relative professional roles, immediate needs and tacit 'rules of play'. In essence, to become empowered actors in the ICU, team members must progress beyond learning procedural steps to understanding the rules of the game: who has power on the team, how is that power commodified, how is it accessed, and in what circumstances is it applied? Understanding these rules can be the difference between knowing how to make something happen in principle (for example, ordering an X-ray) and being able to make it happen in practice (for example, getting an X-ray *now*).

Understanding the rules of the game is also essential if team members are to move beyond thinking as individuals to begin thinking as part of a team.

The purpose of this study was to describe these tacit 'rules of the game'. We sought to determine how power is commodified and exchanged by ICU team members in their daily interactions as they work to achieve clinical goals, delineate professional boundaries, and problem solve around complex system issues.

## Methods

In a follow-up to 4 months of ethnographic non-participant observations (phase 1, detailed methods and results previously reported [9]), seven 1-hour focus groups were conducted with ICU team members in two urban teaching hospitals in Toronto, Canada. Two hospitals were included because the participating intensivists and residents divide their time between the sites and because differences in the settings (for example, case types and case loads, nurse staffing patterns and hospital cultures) might affect team communication and collaboration.

A semi-structured question script was derived to pursue recurrent patterns identified in the observational data. Participants consisted of a sample of four nursing focus groups (*n* = 27), two resident groups (*n* = 6 or 10 available individuals) and one intensivist group (*n* = 4 of 8 available individuals). Residents and intensivists constituted a convenience sample of individuals who were able to accommodate the time for the focus group discussion. Within the nursing group, purposeful sampling was used to ensure some range in years of ICU experience and age in this population [17]. The sample was selected through consultation with the nurse managers of the units. The number of focus groups conducted was determined through theoretical sampling, in which data collection occurred alongside preliminary analysis, and collection ceased when no new themes were arising from the focus group discussions [18]. The study received institutional ethics approval, and informed consent was obtained from all participants.

Focus group interviews were audio-recorded, anonymized and transcribed with standard linguistic conventions to yield about 140 pages of transcription for analysis. In the grounded theory tradition [19], transcripts were read iteratively by four researchers and were analyzed for emergent themes as well as for the themes identified in the analysis of the observational data. Both open coding (identification of primary themes) and axial coding (analysis of relationships among themes) were conducted. The combined expertise of the four analysts was essential to the coding process: one researcher was an intensivist experienced in qualitative research, one was an expert in team discourse, and the remaining two had conducted the observations in the first phase of the study. Emergent themes were revised and refined through the constant comparison of instances from the data set both by individual researchers and in a series of weekly 2-hour meetings during which the analysts compared interpretive memos and discussed relationships between categories. Discrepancies were given particular attention to ensure the validity of the analysis: they were considered by consulting specific instances in the transcripts, discussing their relationship to established themes, and reaching consensus as a group [20].

## Results

The phase 1 observation data provided insight into three areas: the shifting notion of team, the fluctuating levels of collaboration and tension on the team, and the catalysts underlying such fluctuations (previously reported) [9]. Thematic analysis of the focus group data extended our understanding of these three areas, in particular revealing team members' perceptions of the mechanisms by which collaboration is achieved or undermined. Two dominant mechanisms were recurrently described and were categorized in our analysis as 'the perception of ownership' and 'the process of trade'. The findings reported here describe these mechanisms as revealed by the focus group data and supported by the observational data; implications for team collaboration and conflict are emphasized.

### Perception of ownership

This category included references to team members' perceived ownership of valued constructs or commodities, including specialized knowledge, technical skills, equipment, clinical territory and even the patient himself or herself. These constructs and commodities formed the basis of negotiation or exchange during interprofessional interactions. The title of 'ownership' rather than the more traditional concept of 'role' was selected to reflect the participants' emphasis on possession.

Ownership was perceived as both collective (for example, ownership by the ICU team) and individual (for example, ownership by a nurse or by nursing as a profession). Shared perception of collective ownership was portrayed by participants as the foundation of the group's identity. It promoted collaboration between members of the ICU team and was often established by contrast with those outside the core team such as surgeons, internists, or nurses from the wards. For example, nurses explained the team's collective ownership of the patient in contrast to interlopers from outside the unit:

'*We don't negotiate in the ICU because we are ultimately responsible for the patient, so there is no negotiating when you are in charge of that patient*' (Nurse FG1).

Individual ownership was also a dominant issue and included instances where team members recognized their own or others' possession of valued commodities. For instance, respiratory therapists acted in a proprietary manner regarding the ventilator, and this ownership was recognized and respected by other team members. One resident acknowledged that:

'*The RTs' role is probably essential, because, uh, as a medicine resident, we don't know much about the ventilators ... we don't have the time to learn the specifics that they know, so they contribute in areas that we– –we can't...*' (Resident FG1).

In cases like this, the recognition of others' possession of knowledge and skills is part of the smooth collaborative functioning of the team. However, individual ownership can also create interdisciplinary tension when team members feel that their ownership of particular knowledge and skills is not recognized:

Nurse: '*And we're the ones who do keep track because we're there 24 hours a day. It'll be like: "Well order a blood culture", well we did one just yesterday. Or "Order a thyroid test." They just did them 2 days ago. You know?*' (Nurse FG4).

In both observations and focus group data, the designation of ownership was a complex mechanism and frequently a site of tension. In some cases, the allocation of ownership was defended by a particular group and in others, chafed at:

Intensivist: '*At the end of the day the staff [intensivist] is the bottom line. I mean for better or for worse. I am not necessarily saying that it's the right thing but ... the amount of control you relinquish is really wholly dependent on how strong you feel these other members of the team are*' (Intensivist FG1).

Nurse (describing a situation at morning rounds): *'The staff intensivist asked the nurse, are there any issues, any concerns for the patient going to the floor?" The nurse started up, and she was talking about blood pressure issues. The staff intensivist interrupts to say, "Oh well, that's a medical issue. No, I mean specifically a nursing issue. So shot her down immediately*' (Nurse FG2).

The staff intensivist in the first example asserts his ultimate responsibility for patient care. In the latter example, however, the knowledge designated as nursing territory by the intensivist was perceived by the nurse as inappropriately constrained, signaling a conflict between the two professional domains.

Although the recognition of others' ownership of commodities frequently facilitated smooth team function, it also served as a provocation for usurpation and theft. For instance, nurses reported situations in which residents sought nursing knowledge but later portrayed that knowledge as their own:

'*They rely on our notes and our talking to them in the morning to give them the physical assessment of the patient but then they totally disregard you when it comes to rounds as part of the team as though they've done this assessment themselves and nothing you say is worthwhile*' (Nurse FG4).

Participants' discussions of ownership illustrated key problems on an interprofessional team, problems that revolve around respecting the interface between individual and collective knowledge and the balance between individual and collective responsibility.

### Process of trade

This second category captured instances in which team members traded valued commodities as they negotiated their collaborative work. Such trade commonly involved concrete, physical commodities, including equipment and resources, and abstract, social commodities, including respect, goodwill and knowledge.

The trade of scarce physical resources was a catalyst for tension on the team. In many cases, this tension was amplified by its recurrence and by the infuriating smallness of some of the issues under debate:

Nurse: '*I'll give you an example: I need a pump because my patient's blood pressure is dropping and some nurse is hoarding all of them and saying she needs it too. And I say, "I don't think you need it", so I just yank it out and get it because I know this is just a regular drip*' (Nurse FG1).

Trade in such mundane resources was a commonplace ritual as team members negotiated to locate the items required for everyday patient care. In other cases, tension was amplified by the critical importance of the resources. Trade in beds, for example, was fraught with tension, particularly for trainees:

Resident: '*There is always a shortage of nurses and they're always closing beds and we [trainees] sort of have to bear the brunt ... and get caught in a bed war*' (Resident FG1).

Nurse: '*[There was] a new resident on call and the ER calls him, he accepts the patient. And then after he accepts the patient he comes to me to say, "Well, we have a patient", and I say, "No, you don't do that. You ask me first, do we have any beds?" Things like that. They're learning the rules*' (Nurse FG2).

As the latter example illustrates, the trade in physical resources is governed by implicit, social rules, such as who can authorize a trade. Trainees frequently had difficulty in recognizing and negotiating these implicit rules.

Alongside the trade of concrete resources was trade in more abstract commodities. For the nursing group, the most dominant currency for trade was 'respect', which they described themselves expecting in return for information, knowledge, resources and goodwill. The failure of other team members to present the currency of respect was often met with revenge strategies in the form of an embargo of trade. For instance, a nurse might refrain from offering her knowledge if appropriate respect was not proffered first:

Nurse: '*[Consultants to the ICU should] introduce themselves, to say what service they're from, and to ask some questions about the patient as you're the primary caregiver. And ... then they would learn so much more and it would save a lot of time, instead of digging through all this information ... they're flipping, flipping, trying to find bloodwork, but they're not asking me, so I'm not going to help, you know? You find it yourself*' (Nurse FG2).

Such trade of knowledge for goodwill occurred not only among team members but also between the ICU team and consulting teams. This critical sort of trade was recognized and discussed by all team members in the study. Failure to engage in such trade could mean that 'a good team approach was lost' (Nurse FG2). It could also be seriously detrimental to an individual team member's success. For instance, residents expressed that

'*Your name can be ruined or made on one ... encounter, so ... you have to be very careful, because if you create one enemy you can end up having a tough time with a lot of people, and if they love you, then they love you mostly for whatever the time that you're here ... so it's a bit of a social game; you have to be careful*' (Resident FG1).

The process of trade was a constant and at times difficult social game with potentially long-term consequences. The constancy of trade caused it to be a source of accumulated tension and perceived historical injustices, with a single trade event causing a ripple effect that might impact other patients, other team members, other hospital services, or other events later in time. For instance, based on experience, one nurse asserted that

'*When you want to transfer a patient in a hurry there will be an obstruction there ... you know there will be excuses. You know sometimes we feel like they're [ward nurses] prolonging it ... so I say, "Well, I'll call housekeeping for you." Of course they don't like that...*' (Nurse FG1).

The environmental tensions endemic to the ICU served to make the successful negotiation of trade more difficult but also more essential. As one staff intensivist put it:

'... *we deal with a lot of conflict and you have to learn how to control yourself and how to become adept at conflict resolution. And not through intimidation and humiliation of the colleagues you have but honestly listening to them and trying to understand where they are coming from and trying to be respectful of them although ... that is tough sometimes when you are not feeling particularly patient or magnanimous towards these folks that you are talking with and, you know, you are tired, you're sleep deprived ... and you may be getting hassled from all sorts of people because of resource issues*' (Intensivist FG1).

## Discussion

Our data depict team collaboration in a decidedly non-romanticized manner. The notion of team collaboration as rooted in the ownership and trade of commodities presents a stark contrast – and a strong challenge – to the established literature on creating medical teams, which emphasizes mutual support and shared goals, and minimizes competition and contest. What our participants describe as underlying 'rules' in the daily negotiation of individual and team activity, the literature has tended to portray as 'barriers' to teamwork [16]. Recent ethnographic studies of health professional teams suggest that the traditional conception of a stable, unified team does not account for the daily workings of teams in complex environments [6-9]. Further, this current research should caution us that adherence to the traditional ideal of 'team' may, in fact, constrain us from recognizing and promoting the functional mechanisms of group effort in the health care domain.

As our results demonstrate, the forces of ownership and trade have a central role in the daily negotiations that constitute teamwork in the ICU setting. When these forces are ignored – that is, perceived ownership is not attended to, or one commodity is not offered in trade for another – tensions accumulate and collaboration becomes sluggish. When these forces are accommodated – for example, competition for ownership of resources is anticipated, or requests are accompanied by offers of trade – the team members navigate their competing interests more smoothly to act effectively together.

From a sociological perspective, this is common sense. There are sound theoretical reasons for these rituals of ownership and trade, the most basic of which is that the 'team' is not a unified entity but rather a compilation of individuals with distinct professional identities: intensivist, nurse, respiratory therapist, resident, and so on. These professional identities are based in distinct models of care, different skill sets, diverse economic circumstances and competitive political agendas.

A useful way of theorizing the construct of professional identity, particularly when diverse professions come in contact with one another, can be found in the theory of social structuration [21]. In this theory, professions or organizations are conceptualized as social systems, in which each professional's role is determined by its position in relation to others and by its access to certain commodities. These commodities include access to material resources ('economic capital'), access to levels of information ('cultural capital') and access to social connections and acknowledged forms of expertise ('social capital'). Structuration theory is especially useful because it recognizes that individuals both within a profession (such as nursing) and between professions (such as nursing and critical care medicine) are in the constant process of attempting to distinguish themselves and their profession and thus acquire more 'capital' so as to promote their ability to act ('agency') [22]. This notion of a profession and of an interprofessional team as a contested space is important, as it moves beyond a simplified notion of 'community' as a group with shared values [23,24] and allows us to theorize about important tensions in the formation of professional identity and the interaction between multiple professions. Acknowledging these tensions enables us to understand the way in which teams sustain the delicate balance between achieving a shared goal and competing for agency and status in the interprofessional setting.

The forces of ownership and trade are products of the contested relations on an interprofessional team. The point is not to stamp out these forces or to overcome them, but rather to articulate their role in team collaboration, so that they can be more strategically harnessed by team members and, as a consequence, smooth team functioning can be promoted. Handled adeptly, these forces allow members of a team to get necessary clinical work done, even in the chaos of competing ambitions and interests that is the ICU team. As one nurse put it: 'It may be construed that you are demanding, but then if you don't demand sometimes you don't get it; it's just a matter of strategy' (Nurse FG1).

## Limitations

This study is constrained by the design decisions underpinning it. Findings may reflect the attitudes of a subset of ICU team members, for instance those more interested in exploring these topics. Generalizability is not the goal of grounded theory research, which seeks instead to produce rich descriptions and theoretical explanations of situated processes. However, the explanatory utility of these findings may be explored and enhanced in future research in different centers or other interprofessional health care team contexts.

## Conclusions

It is time that our understanding of team collaboration moved beyond the rhetoric of cooperation, and towards a more authentic depiction of the skills required to function in the competitive setting of the interprofessional health care team. Our intention is not to suggest a new rhetoric (of economics), but rather to shift our attention from idealized or abstracted depictions of teamwork, towards a grounded understanding of how collaboration is accomplished in daily practice. Knowing about perceptions of ownership, valued commodities and the rules of trade allows team members to shape outcomes and persuade people, to anticipate reactions and deflect obstructions, and to achieve individual goals while maintaining team cohesion. Efforts to improve teamwork must reflect such authentic, everyday 'rules of the game' if they are to affect how work gets done on health care teams in complex settings such as the ICU.

These findings suggest educational implications relevant both to trainees and practising intensivists. In most training programs, professionalism and collaboration are part of an implicit, ad hoc curriculum largely consisting of role modeling and trial and error. As medical schools respond to recent calls to ensure competence in domains such as communication and collaboration [25], an understanding of authentic collaborative practice is essential to inform evidence-based curricula. For practising intensivists who may experience tension and difficulty in some team situations, understanding the rules of the game may assist them to analyze and improve their collaborative practice and, it is hoped, to improve the quality of care they provide to critically ill patients.

## Contributors

All authors contributed to the design, conduct, analysis and interpretation of the research reported. LL and LH were Co-Principal Investigators and led the conceptual design of the study. SE and CE assisted with data collection and analysis, and with manuscript preparation.

## Key messages

• 	In the daily negotiations that constitute inter-professional ICU teamwork, the ownership and trade of valued commodities play a central role.

• 	When ownership and trade are appreciated and handled well, team members are able to anticipate reactions, deflect obstructions, and achieve individual goals while maintaining team cohesion.

• 	Articulation of such authentic "rules of the game" is essential to the development of evidence-based curricula in collaborative practice.

## Competing interests

None declared.

## Abbreviations

ICU = intensive care unit.
